# A pilot controlled trial of insulin-like growth factor-1 in children with Phelan-McDermid syndrome

**DOI:** 10.1186/2040-2392-5-54

**Published:** 2014-12-12

**Authors:** Alexander Kolevzon, Lauren Bush, A Ting Wang, Danielle Halpern, Yitzchak Frank, David Grodberg, Robert Rapaport, Teresa Tavassoli, William Chaplin, Latha Soorya, Joseph D Buxbaum

**Affiliations:** Seaver Autism Center for Research and Treatment, Icahn School of Medicine at Mount Sinai, One Gustave L Levy Place, Box 1230, New York, NY 10029 USA; Friedman Brain Institute, New York, NY USA; Mindich Child Health Institute, New York, NY USA; Departments of Psychiatry, New York, NY USA; Departments of Pediatrics, New York, NY USA; Departments of Neuroscience, New York, NY USA; Departments of Neurology, New York, NY USA; Departments of Genetics and Genomic Sciences, New York, NY USA; Departments of Endocrinology and Diabetes, New York, NY USA; Icahn School of Medicine at Mount Sinai, New York, NY USA; Department of Psychology, St John’s University, New York, NY USA; Department of Psychiatry, Rush University Medical Center, Chicago, IL USA

## Abstract

**Background:**

Autism spectrum disorder (ASD) is now understood to have multiple genetic risk genes and one example is *SHANK3. SHANK3* deletions and mutations disrupt synaptic function and result in Phelan-McDermid syndrome (PMS), which causes a monogenic form of ASD with a frequency of at least 0.5% of ASD cases. Recent evidence from preclinical studies with mouse and human neuronal models of *SHANK3* deficiency suggest that insulin-like growth factor-1 (IGF-1) can reverse synaptic plasticity and motor learning deficits. The objective of this study was to pilot IGF-1 treatment in children with PMS to evaluate safety, tolerability, and efficacy for core deficits of ASD, including social impairment and restricted and repetitive behaviors.

**Methods:**

Nine children with PMS aged 5 to 15 were enrolled in a placebo-controlled, double-blind, crossover design study, with 3 months of treatment with IGF-1 and 3 months of placebo in random order, separated by a 4-week wash-out period.

**Results:**

Compared to the placebo phase, the IGF-1 phase was associated with significant improvement in both social impairment and restrictive behaviors, as measured by the Aberrant Behavior Checklist and the Repetitive Behavior Scale, respectively. IGF-1 was found to be well tolerated and there were no serious adverse events in any participants.

**Conclusions:**

This study establishes the feasibility of IGF-1 treatment in PMS and contributes pilot data from the first controlled treatment trial in the syndrome. Results also provide proof of concept to advance knowledge about developing targeted treatments for additional causes of ASD associated with impaired synaptic development and function.

## Background

Autism spectrum disorder (ASD) is currently a diagnosis established by behavioral criteria but its etiology resides in complex genetics, and many distinct genetic risk genes have been identified as causal. One such example is the *SHANK3* gene located on the terminal end of chromosome 22q where loss of one copy (haploinsufficiency) of *SHANK3* causes a monogenic form of ASD with a frequency of at least 0.5% of ASD cases [1–4]. The *SHANK3* gene codes for a protein that plays a critical role in synaptic function by scaffolding the postsynaptic density of glutamatergic synapses [5] and its loss is sufficient to cause Phelan-McDermid syndrome (PMS) [6, 7]. PMS is characterized by significant intellectual disability (ID), severely delayed or absent language, and ASD. Affected individuals also suffer from a heterogeneous array of dysmorphic and medical features, including increased risk of seizures, recurring infections, hypotonia, renal disease, and gastroesophageal reflux [8–17]. While PMS accounts for a relatively small proportion of ASD cases, the *SHANK3* pathway is relevant to many forms of ASD because different genetic causes of ASD converge on several common pathways, including *SHANK3*[18, 19].

Using *Shank3*-deficient mice, deficits in synaptic function and plasticity have been shown using electrophysiological measures [20]. Behaviorally, *Shank3-*deficient mice display motor deficits, less social sniffing and reduced ultrasonic vocalizations as compared to wildtype controls [20, 21]. Insulin-like growth factor-1 (IGF-1) has been studied in this mouse, as well as in human neuronal models of PMS, and was found to reverse synaptic and behavioral deficits [21]. Importantly, IGF-1 is also effective in reversing phenotypic changes in human neuronal models of Rett syndrome [22], providing additional evidence that this pathway may be a target in diverse forms of ASD. These data are promising and provide preclinical evidence that IGF-1 may produce disease-modifying effects in subjects with PMS or Rett syndrome. Recent evidence also provides preliminary support of efficacy with IGF-1 in children with Rett syndrome [23].

IGF-1 is a commercially available compound that crosses the blood-brain barrier (BBB) and has beneficial effects on synaptic development by promoting neuronal cell survival, synaptic maturation, and synaptic plasticity [23, 24]. IGF-1 enters the brain from the circulation where it is released mainly by the liver upon growth hormone stimulation. Blood-borne IGF-1 is found in the central nervous system (CNS) where it promotes brain vessel growth [25], neurogenesis, and synaptogenesis [26]. The mechanisms that trigger IGF-1 entry through the BBB are proposed to occur by diffusible messengers released in areas of neuronal activity which lead to cleavage of IGF binding protein-3 (IGFBP-3) to increase free IGF-1 and allow passage of circulating IGF-1 into the CNS [27]. The recent IGF-1 study in Rett syndrome examined its pharmacokinetic profile in human subjects and found that IGF-1 reached the CNS compartment as demonstrated by significantly increased cerebrospinal fluid levels after treatment [23]. However, the precise mechanisms by which IGF-1 exerts its effects on the CNS remains an active area of study.

IGF-1 is currently approved by the Food and Drug Administration (FDA) for the treatment of short stature due to primary IGF-1 deficiency (Laron’s Dwarfism). There have been no controlled treatment trials in PMS to date and this study was designed to evaluate the safety, tolerability, and efficacy of IGF-1 versus placebo in children with PMS targeting core domains of ASD. Specifically, social deficits and repetitive behaviors were measured using the Aberrant Behavior Checklist-Social Withdrawal (ABC-SW) subscale as the primary outcome measure [28] and the Repetitive Behavior Scale-Revised (RBS-R) as a secondary outcome [29].

## Methods

All subjects were recruited under an Institutional Review Board (IRB) approved protocol as part of ongoing studies in PMS at the Seaver Autism Center for Research and Treatment at the Icahn School of Medicine at Mount Sinai and parents/guardians provided informed consent. Treatment followed a randomized, placebo-controlled, crossover format with 12 weeks in each treatment arm (IGF-1 and placebo), separated by a 4-week wash-out phase (ClinicalTrials.gov Identifier: NCT01525901). This was a pilot study in a rare disorder, PMS, and sample size and analyses were defined *a priori.* Multiple outcome measures are being collected as part of a larger ongoing clinical trial with IGF-1 in PMS but pilot data from the safety measures, and main primary and second outcomes have been analyzed to date and are presented herein.

### Inclusion criteria

This pilot recruited 9 children between 5 and 15 years-old with PMS and confirmed to have *SHANK3* deletions or mutations based on chromosomal microarray or high-throughput or targeted sequencing. All subjects were on stable medication regimens for at least 3 months prior to enrollment.

### Exclusion criteria

Cases were excluded if any of the following were applicable: 1) closed epiphyses; 2) active or suspected neoplasia; 3) intracranial hypertension; 4) hepatic insufficiency; 5) renal insufficiency; 6) cardiomegaly/valvulopathy; 7) allergy to IGF-1; 8) patients with comorbid conditions deemed too medically compromised to participate.

### Drug administration

IGF-1 (Increlex; Ipsen Biopharmaceuticals, Inc) is an aqueous solution for injection containing human insulin-like growth factor-1 (rhIGF-1) produced by recombinant DNA technology. Placebo consisted of saline prepared in identical bottles by the research pharmacy at Mount Sinai. We received an Investigational New Drug exemption from the Food and Drug Administration (#113031) to conduct this trial in children with PMS. Based on the package insert for Increlex, dose titration was initiated at 0.04 mg/kg twice daily by subcutaneous injection, and increased, as tolerated, every week by 0.04 mg/kg per dose to a maximum of 0.12 mg/kg twice daily. This titration was justified based on our preclinical data, which indicated that 0.24 mg/kg/day is effective in reversing electrophysiological deficits whereas 0.12 mg/kg/day was not as effective [21]. We aimed to reach the therapeutic dose as quickly as is safe and tolerated in order to allow maximum time for clinical improvement. Doses could be decreased according to tolerability by 0.04 mg/kg per dose. Medication was administered twice daily with meals, and preprandial glucose monitoring was performed by parents prior to each injection throughout the treatment period. Parents were carefully trained in finger stick monitoring, symptoms of hypoglycemia, and medication administration.

### Outcome measures

Efficacy measurements were taken at baseline of each treatment phase, and at weeks 4, 8, and 12 of each treatment phase. The ABC is a rating scale used to monitor an array of behavioral features, including social withdrawal (i.e., Lethargy subscale). The ABC-SW subscale was chosen *a priori* as the primary outcome measure because it is well validated in both ID and ASD patients and is currently accepted as an appropriate outcome measure within the field of pediatric psychopharmacology research. In addition, preliminary data suggest that the ABC accurately reflects ASD-related deficits in PMS. Repetitive behavior was measured using the RBS-R, which has also been previously validated as a measurement tool in ID and ASD populations. Safety, tolerability, and adverse events (AEs) were measured throughout the trial during monitoring visits and phone calls using an adapted semi-structured interview, the Safety and Monitoring Uniform Report Form (SMURF) every 2 weeks, and extensive clinical and laboratory assessments every 4 weeks. AEs were documented with respect to severity, duration, management, relationship to study drug, and outcome.

### Data analysis

This trial used a randomized crossover design; thus, order (phase) of assessment is nested (repeated) within treatment and treatment is nested within subjects. Given the small sample size, we considered several analytic strategies and selected a fully within-subject, repeated-measures design. To conform to intent-to-treat principles, we used mixed effects regression models so that participants missing observations would be included in the analysis (full information maximum likelihood estimation). Treatment was dummy coded as 0 = placebo and 1 = control and time coded naturally as weeks (0, 4, 8, and 12). In this analysis, the primary focus was time by treatment interaction, which estimates the differential change in the two treatments on the outcome measures. Because of the small sample size, we used a variance components intercept only model for the random effect variation. We then added ‘phase’ as another repeated factor to examine the effect of order through a three-way interaction analysis.

## Results

A total of 9 children and adolescents (6 females and 3 males) were screened based on the established diagnosis of PMS and all 9 were randomized to treatment order. Subjects ranged in ages from 5 to 15 years old at the time of entry into the study (X = 8.6 years; SD = 4.0). At baseline, the mean ABC-SW subscale score was 16.9 (SD = 8.7) for all subjects and the mean RBS-R Total score was 32.1 (SD = 23.5). Cognitive abilities were evaluated with the Mullen Scales for Early Learning [30] or the Leiter International Performance Scale-Revised [31] and adaptive functioning with the Vineland Adaptive Behavior Scales, Second Edition [32]. All participants met criteria for ID based on mental age equivalent scores on IQ measures and adaptive behavior composite scores (see Table 1). The Leiter was the preferred measure of nonverbal IQ in our sample given the potential to calculate standard scores in this age range. However, only two of nine participants had the pre-requisite skills to participate in a Leiter administration based on the ability to make a two-part discrimination, sit, and attend. For the remaining 7 of 9 participants, we used the Mullen despite the fact that the upper end of the Mullen standardization sample is 68 months. All subjects met criteria for ASD according to expert clinical consensus and results from the Autism Diagnostic Observation Schedule-Generic [33], the Autism Diagnostic Interview-Revised (ADI-R; [34]), and the *Diagnostic and Statistical Manual for Mental Disorders-5*[35].

**Table 1:** **Baseline demographic characteristics**

Subject	Chronological age (months)	Estimated mental age equivalent (months)	Vineland adaptive behavior scale composite standard score
1	177.4	8.5	29
2	103.7	10.3	47
3	66.3	11.3	52
4	64.6	12.3	43
5	109.7	^a^36	61
6	91.8	7	50
7	172.7	30.5	31
8	71.1	^a^31	51
9	61.8	9.3	45

^a^Denotes scores from the Leiter International Performance Scale-Revised; all other scores were based on the Mullen Scales for Early Learning.

Because this was a small pilot trial, our primary focus was on safety. IGF-1 was generally well tolerated and there were no serious adverse events (SAEs) (see Table 2). The most common side effects during the IGF-1 treatment phase were sleep disturbance (N = 7); hypoglycemia ((<50 mg/dL) (7 occurrences; N = 5)), constipation (N = 4), increased appetite (N = 4), and mood changes or increased irritability (N = 4). Other less common AEs ‘possibly’ or ‘probably related’ to IGF-1 and worth noting included decreased appetite (N = 2), increased urinary frequency (N = 1), hair loss (N = 1), rash (N = 1), and soft tissue swelling of the nose (N = 1). The rash resolved upon discontinuing IGF-1 and did not recur upon re-challenge. The soft tissue swelling also resolved upon completion of the treatment phase without intervention or dose adjustment. One patient had to be discontinued because they developed an upper respiratory tract infection requiring antibiotics that led to gastrointestinal symptoms, constipation, and decreased appetite and they could not adequately sustain their glycemic state to tolerate treatment. At the early termination visit, the patient was fully recovered without sequelae. No other patients required dose adjustments during the trial. Many of the same AEs were likely to occur among patients during the placebo phase, including hypoglycemia (3 occurrences; N = 2), albeit less frequently; significantly more AEs were reported when patients were taking IGF-1 than placebo (*P* = 0.0015). There were no significant changes in growth parameters, including height, weight, head circumference, or bone age over the course of the trial.

**Table 2:** **Adverse events**

Adverse events	IGF-1	Placebo
Constipation	4	3
Sedation	1	0
Decreased appetite	2	3
Periobital/facial swelling	1	0
Diarrhea	1	2
URTI^a^	5	5
Sleep Disturbance	7	2
Increased appetite	4	0
Mood changes/irritability	4	2
Increased thirst	1	0
Increased phlegm	1	0
Teeth grinding	1	0
Cough	1	2
Hand flapping	1	0
Increased bowel movements	1	0
Increased chewing/biting	1	0
Decreased visual acuity	1	0
Lethargy/decreased energy	1	1
Cooler body temperature/sweating	1	0
Runny nose/congestion	1	1
Gait changes/fell	1	2
Stomach virus	1	1
Anxiety	0	2
Increased urine frequency	2	0
Fever	3	3
Increased energy	1	0
Gagging	1	0
Increased thirst	0	1
Conjunctivitis	1	0
Erythema/swollen eyes	1	0
Vomiting	1	1
Rash	3	0
Nose swelling	1	0
Warmer body temperature	1	0
Hair loss	1	0
Increased aggression	1	0
Hypoglycemia^b^	7	3

^a^URTI = upper respiratory tract infection. ^b^Hypoglycemia was defined by glucose < 50 mg/dL and occurred in 5/9 patients while on IGF-1 and in 2/9 patients while on placebo.

**Figure 1:**
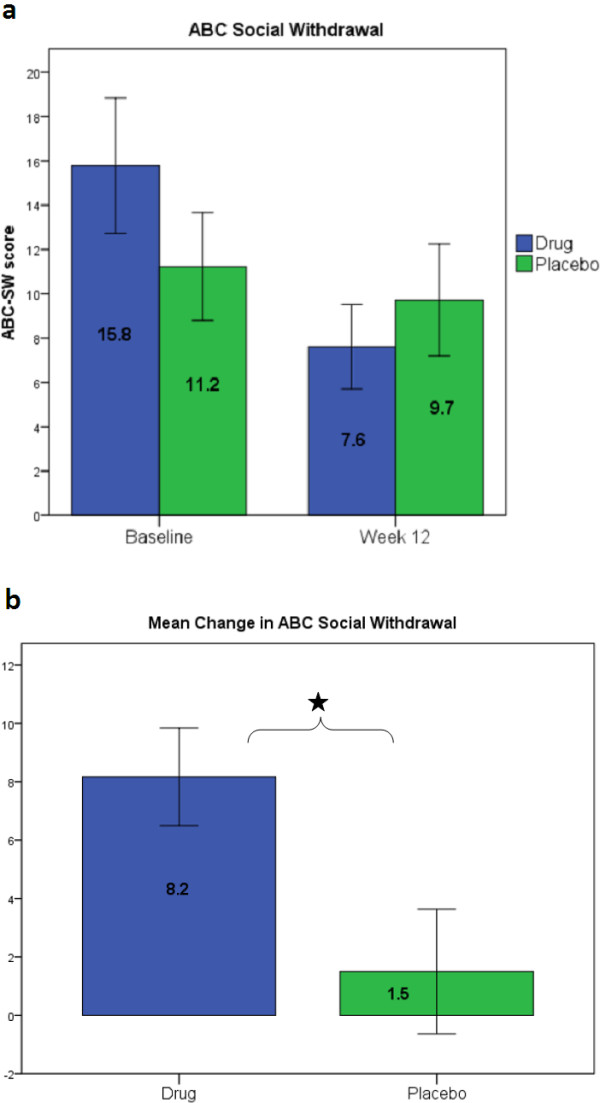
**ABC-SW score between baseline and week 12 of drug or placebo. (A)** Change in ABC-SW score between baseline and week 12 of drug or placebo for 9 subjects. In this and all subsequent figures, treatment condition (drug versus placebo) was combined across treatment phases. Error bars = ±1 SE. **(B)** Mean change in ABC-SW score between baseline and week 12 of drug or placebo. *P* = 0.040; error bars = ±1 SE. ABC-SW = Aberrant Behavior Checklist Social Withdrawal subscale; SE = standard error.

**Figure 2:**
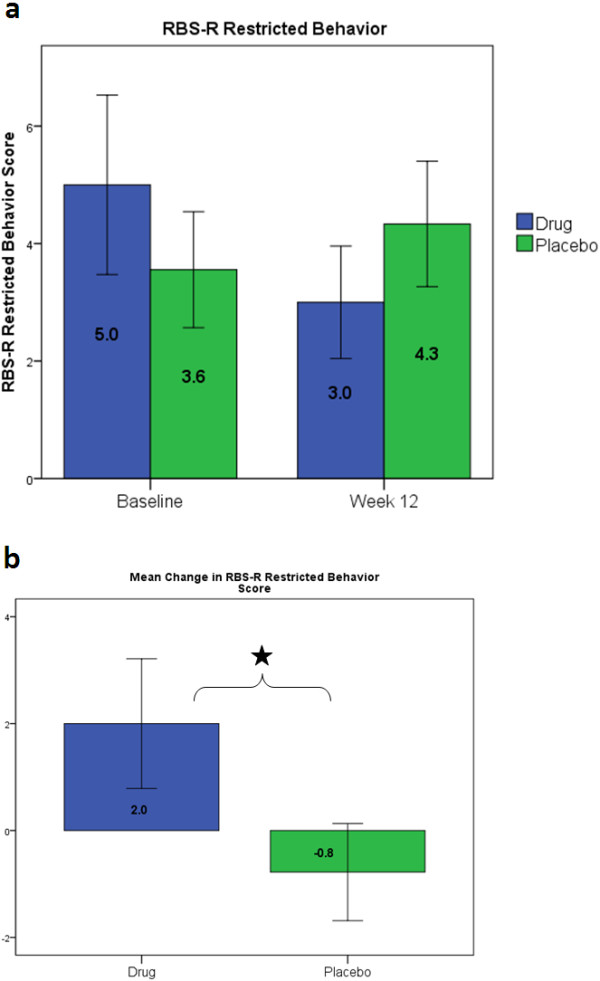
**RBS-R Restricted Behavior score between baseline and week 12 of drug or placebo. (A)** Change in RBS-R Restricted Behavior score between baseline and week 12 of drug or placebo. **(B)** Mean change in RBS-R Restricted Behavior score between baseline and week 12 of drug or placebo. *P* = 0.042; error bars = ±1 SE. RBS-R = Repetitive Behavior Scale-Revised. SE = standard error. The star reflects the p value and notes the significance.

In terms of efficacy, when participants received IGF-1 they showed significant improvement as compared to when they were on placebo (see Figures 1 and 2). There was a significant two-way time x group interaction across both phases on the ABC-SW subscale (N = 9; t = −2.107; *P* = 0.040). Importantly, when ‘phase’ (order) was added as a third variable, the three-way interaction of group x time x phase was not significant. This indicates that the time x group interaction is not significantly different as a function of treatment order and the two-way interaction is a valid reflection of the drug effect. Inspection of a longitudinal plot of the data suggested that the drug condition showed improvement in both phases whereas the placebo group showed little change in either phase.

However, participants assigned to IGF-1 at phase 1 had a higher baseline ABC-SW value at phase 1 than the placebo participants. Given the small sample size, this disparity is not surprising despite randomization. To account for this disparity we controlled for phase 1 ABC-SW baseline values and the time x treatment interaction remained significant (N = 9; t = −2.151; *P* = 0.036). Analyses were performed on the other ABC subscales and results were not statistically significant (Irritability: t = −0.839, *P* = 0.405; Motor Stereotypies: t = −0.240, *P* = 0.811; Hyperactivity: t = −0.954, *P* = 0.344; Inappropriate Speech: t = 0.008, *P* = 0.994).

Results from the RBS-R Total score were not significant (N = 9, t = −0.791; *P* = 0.432). However, analyses were performed on the RBS-R subscales and significant improvement was found on the Restricted Behavior subscale (N = 9; t = −2.077; *P* = 0.042). Again, when ‘phase’ (order) was added as a third variable, the three-way interaction was not significant indicating that the significant time x group interaction is not a function of treatment order. Participants also had a higher baseline RBS-R Restrictive Behavior scores at phase 1 than the placebo participants, but after adding the baseline values of the RBS-R Restricted Behavior subscale to the model, the interaction remains significant and is largely unchanged (t = −2.107; *P* = 0.040). Results from the other RBS-R subscales were not statistically significant (Stereotyped Behavior: t = −0.269, *P* = 0.789; Self-Injurious Behavior: t = −1.896, *P* = 0.063; Compulsive Behavior: t = 0.888, *P* = 0.378; Ritualistic Behavior: t = −0.192, *P* = 0.848; Sameness: t = 0.005, *P* = 0.996).

## Discussion

This is the first controlled treatment trial in PMS and establishes safety and preliminary evidence of benefit of IGF-1 on core symptoms of ASD. Although PMS is now recognized to be a relatively common cause of ASD, a review of the literature revealed only two published reports of medication treatment trials, one case series with intranasal insulin [36] and one case study with risperidone [37], both open label and with reported improvement. In the intranasal insulin study, 6 children received up to 12 months of treatment with improvement based on sum of scores from a parental questionnaire in most domains, including cognitive, speech and language, and motor skills [36]. Insulin was hypothesized in these cases to improve neuronal function by increasing CNS glucose uptake and enhancing synaptic plasticity via glutamatergic receptors. Results from the case study with risperidone were similarly optimistic but equally uncontrolled: an 18-year-old girl with PMS was treated with risperidone 0.5 mg twice daily with significant improvement on the Clinical Global Impression Scale (CGI; [38]) in anxiety, aggression, and insomnia after 1 month that was sustained after 6 months. The hypothesized mechanism of action of risperidone in *SHANK3* deficiency was to promote N-methyl-D-aspartate (NMDA) transmission via dopamine 2 receptor blockade. However, studies in *Shank3* deficient mice indicate that reduced basal neurotransmission at glutamatergic synapses may be α-amino-3-hydroxy-5-methyl-4-isoxazolepropionic acid (AMPA) receptor mediated [20].

To date, few pharmacological treatments in ASD have been developed based on neurobiological strategies. Most trials have employed etiologically heterogeneous samples and targeted associated symptoms. More recently, genetic discovery and model systems have led to important opportunities for developing novel, potentially disease-modifying therapeutics. This study is based on preclinical evidence showing that IGF-1 reversed deficits in mouse and neuronal models of *SHANK3* deficiency. The results now provide preliminary evidence of efficacy with IGF-1 in a clinical sample of children with PMS. Recent examples of a similar approach to treatment development are the large-scale clinical trials currently underway in fragile X syndrome (FXS), Rett syndrome, and tuberous sclerosis, all known causes of ASD. The *SHANK3*/glutamate signaling pathway is highly relevant to various forms of ASD [18, 19] and the link between deficits in synapse function and ASD suggest that treatment with IGF-1 may have implications for ASD caused by disruptions in common underlying pathways. Importantly, the first evidence of the efficacy of IGF-1 in an ASD-related syndrome has already emerged in Rett syndrome [23].

Despite the promise of these findings, several weaknesses must be acknowledged. For example, the small sample challenges our ability to generalize results to a broader population of children with PMS. The resulting decrease in statistical power did not compromise the significance of our findings but it is possible that the effect size may not be upheld in a larger sample. In addition, we showed that when patients received IGF-1 they were significantly more likely to report AEs and this may have introduced bias and contributed to the caregivers’ anticipation of benefit. Such challenges are inherent to using caregiver reports as outcome measures. Further, while patients were more likely to report AEs while on IGF-1, the nature of AEs was similar between treatment groups. Lastly, crossover trial designs carry risks of treatment order effects. We provided a 4-week wash-out period to minimize this risk and evaluated the differential effect of treatment over time in the context of order to show that there was no significant effect of treatment order. We chose to use this design because PMS is a rare disease without available treatments and wanted to ensure that all participants were afforded the opportunity to receive active medication; the cost of IGF-1 prohibited an open label extension trial.

## Conclusions

Results from this study are novel and of broad interest for a number of important reasons. First, PMS, while considered a rare disorder, is now known to be a more common, and highly penetrant cause of ASD [39]. As such, there is significant interest in the *SHANK3* gene as genetic discovery and functional analysis of model systems continue to aid in our understanding of the neurobiology of ASD. Second, this is a translational clinical trial based on prior work in mice that demonstrates IGF-1 to reverse electrophysiological and motor learning deficits [21]. Third, an independent group has demonstrated a reversal of synaptic deficits with IGF-1 in neurons derived from induced pluripotent stem cells of patients with PMS [21]. Fourth, there is now additional independent clinical evidence of the efficacy of IGF-1 in Rett syndrome to generate interest in this compound. Finally, we have shown benefits with IGF-1 on core symptoms of ASD, suggesting a potentially disease-modifying effect. Of course, these positive findings must be interpreted with caution given the small sample size. With its limitations, the present study nevertheless contributes the first evidence of the safety and efficacy of IGF-1 in PMS from the first controlled treatment trial in the field. Results from this pilot are intended to facilitate larger studies that will more definitively inform efficacy.

## Abbreviations

ABC: Aberrant Behavior Checklist
ADI-R: Autism Diagnostic Interview-Revised
ADOS-G: Autism Diagnostic Observation Schedule-Generic
AE: adverse event
AMPA: α-amino-3-hydroxy-5-methyl-4-isoxazolepropionic acid
ASD: autism spectrum disorder
BBB: blood-brain barrier
CGI: Clinical Global Impression Scale
CNS: central nervous system
*DSM-5*: *Diagnostic and Statistical Manual for Mental Disorders*
FDA: Food and Drug Administration
ID: intellectual disability
FXS: fragile X syndrome
IGF-1: insulin-like growth factor-1
IGFBP-3: IGF binding protein-3
IRB: Institutional Review Board
NMDA: N-methyl-D-aspartate
PMS: Phelan-McDermid syndrome
rhIGF-1: recombinant human insulin-like growth factor-1
RBS-R: Repetitive Behavior Scale-Revised
SAE: serious adverse event
SMURF: Safety and Monitoring Uniform Report Form.
