# Erratum: A pilot controlled trial of insulin-like growth factor-1 in children with Phelan-McDermid syndrome

**DOI:** 10.1186/s13229-015-0025-0

**Published:** 2015-06-02

**Authors:** Alexander Kolevzon, Lauren Bush, A Ting Wang, Danielle Halpern, Yitzchak Frank, David Grodberg, Robert Rapaport, Teresa Tavassoli, William Chaplin, Latha Soorya, Joseph D Buxbaum

**Affiliations:** Seaver Autism Center for Research and Treatment, Icahn School of Medicine at Mount Sinai, One Gustave L Levy Place, Box 1230, New York, NY 10029 USA; Friedman Brain Institute, New York, NY USA; Mindich Child Health Institute, New York, NY USA; Departments of Psychiatry, New York, NY USA; Departments of Pediatrics, New York, NY USA; Departments of Neuroscience, New York, NY USA; Departments of Neurology, New York, NY USA; Departments of Genetics and Genomic Sciences, New York, NY USA; Departments of Endocrinology and Diabetes, New York, NY USA; Icahn School of Medicine at Mount Sinai, New York, NY USA; Department of Psychology, St John’s University, New York, NY USA; Department of Psychiatry, Rush University Medical Center, Chicago, IL USA

## Erratum

After the publication of [1], the authors noticed that a reference had been omitted from the text and reference list.

In the introduction and conclusion sections, there are references to using IGF-1 in human neuronal models of Phelan-McDermid syndrome. The correct reference for this study is:

Shcheglovitov A, Shcheglovitova O, Yazawa M, Portmann T, Shu R, Sebastiano V, Krawisz A, Froehlich W, Bernstein JA, Hallmayer JF, Dolmetsch RE. SHANK3 and IGF1 restore synaptic deficits in neurons from 22q13 deletion syndrome patients. Nature. 2013 Nov 14;503(7475):267–71. doi: 10.1038/nature12618. Epub 2013 Oct 16. PubMed PMID: 24132240
